# Incontinence in the Elderly

**Published:** 1992-12

**Authors:** G. W. Tobin


### Incontinence in the Elderly

**Dr. G. W. Tobin, M.R.C.P**

The International Continence Society defines urinary incontinence as a condition in which the involuntary loss of urine is a social or hygienic problem and is objectively demonstrable. There is wide variation in the reported prevalence of incontinence in the elderly. This is due mainly to the different definitions of incontinence used in different studies. In one widely quoted survey (*Thomas et al* 1980) the authors defined incontinence as the involuntary loss of urine on two or more occasions per month. Using this definition they found the prevalence of incontinence in the elderly to be 11.6% in women and 6.9% in men. The prevalence of incontinence is higher still among those in institutional care. 30% of those in residential care, 50% of patients in nursing homes, 70% of patients in continuing care geriatric beds and 80% of patients in long stay psychogeriatric wards, are incontinent of urine. These high levels presumably reflect the advanced age of these populations and the high prevalence of neurological disorders.

Over half of the incontinent elderly are unknown to the medical or nursing services. This under reporting occurs for many reasons. Some patients, especially females, believe that incontinence is a normal consequence of aging. Others are afraid of being pressurised into having an operation. Many are simply too embarrassed to admit to having a problem with continence.

The psychological and social morbidity caused by incontinence bears no relationship to it's severity. In general patients with urge incontinence suffer greater psychological stress than those with stress incontinence. Studies have shown significantly higher levels of anxiety and depression among those who are incontinent. They have reduced contact with others, and restrict their social activities (*Macauley et al* 1987).

### PATHOPHYSIOLOGY

Urinary incontinence in the elderly can be divided into transient and established incontinence. Transient incontinence is said to account for a third of the incontinence among the elderly in the community and half of the incontinence seen in acutely ill elderly patients admitted to hospital. It is usually associated with a toxic confusional state. The incontinence resolves as the confusion resolves. Transient incontinence may also be associated with faecal impaction, impaired mobility and various drugs, e.g., diuretics, sedatives.

The unstable detrusor,.defined by the International Continence Society as one which contracts spontaneously or on provocation during the filling stage of a urodynamic study while the patient is attempting to inhibit micturition, is the commonest cause of established incontinence in the elderly. The definition used to require a pressure rise of greater than 15 cm of water. Although a specific value is no longer required to make the diagnosis it is implicit in the definition that the pressure rise should be phasic. These involuntary contractions may or may not be associated with urine loss. Patients may complain of urinary frequency, nocturia, urgency, urge incontinence or stress incontinence. Urgency associated with an overactive detrusor is referred to as motor urgency. Urgency and urge incontinence may also occur in patients with hypersensitive bladder conditions, when they are referred to as sensory urgency and sensory urge incontinence. Although incontinence is common in patients with motor urgency it is much less common in sensory urgency.

When detrusor overactivity is due to dysfunction of the neural control of the bladder the term detrusor hyperreflexia is used. A detailed description of neuropathic bladder disorders is beyond the scope of this paper. The common causes in the elderly are cerebrovascular disease, dementia and Parkinson's disease. These conditions, not only cause incontinence but also act as aggravating factors by impairing patients mobility, cognition and communication. Although incontinence is common in the early stages of a stroke, it is usually transient. Only 15 - 30% of survivors remain incontinent six months following a stroke. Incontinence is of prognostic value in patients who have had a stroke. Less than 3% of those continent after a stroke will die. Of those who are incontinent but regain continence in the first month, 80% will return to the community.

In elderly males the unstable detrusor may occur secondary to outflow tract obstruction, usually due to prostatic hyperplasia. 50% of men with significant outflow obstruction have an unstable detrusor. The occurrence, though not of the severity of bladder instability correlates with the degree of outflow obstruction (*Cucchi* 1988). Around 2/3 of patients will regain continence after surgery to relieve the outflow obstruction. The number of men with persistent instability after relief of outflow obstruction approximates to the number of women of the same age who have detrusor instability. Outflow tract obstruction is much rarer in elderly females and is not associated with instability. By definition the cause of idiopathic detrusor instability is unknown. In younger age groups it is often considered a psychosomatic condition. Some elderly patients with detrusor instability who have no evidence of outflow obstruction or neurological disorder may have sub clinical neurological damage.

Another common cause of established incontinence in elderly females is Genuine stress incontinence. This is defined as the involuntary loss of urine when the intravesical pressure exceeds maximum urethral pressure due to elevation of intra abdominal pressure and in the absence of detrusor contraction. It is usually due to failure of the anatomical support of the bladder and urethra. Because of defects in the support of the urethra and bladder neck the urethra becomes an extra abdominal organ either at rest, or when intra-abdominal pressure is raised. Rises in intra-abdominal pressure are transmitted to the bladder raising intravesical pressure but are not transmitted efficiently to the proximal urethra. There is a transient reversal of the normal pressure gradient with intravesical pressure now exceeding intra-urethral pressure. The result is loss of urine. In the situation just described the sphincter mechanism itself is normal and can maintain a watertight seal. Incontinence occurs because it is in an abnormal position. In some patients the sphincter mechanism can no longer maintain a watertight seal. This is referred to as type III stress incontinence. The importance of this condition is that it will not respond to the usual operations for Genuine stress incontinence which aim to restore the bladder neck and urethra to their normal position. Correction of the compression defect requires the use of urethral sling procedures.

There are other, less common, causes of incontinence in the elderly, these include:

- Fistulae which in developed countries are usually a complication of gynaecological surgery, urological surgery, bowel surgery or radiotherapy. They may also be malignant.
- The unstable urethra, a poorly understood condition in which there are fluctuations in urethral pressure.
- Functional incontinence in which patients are incontinent despite a normally functioning lower urinary tract, e.g., depression.

Many elderly patients will be found to have more than one pathophysiological mechanism for their incontinence. Even where there is a single pathophysiological mechanism, it may be difficult to know which of two different diseases is causing it. An elderly man with urge incontinence due to detrusor over activity may have Parkinson's disease and prostatic hyperplasia. He may have detrusor hyperreflexia secondary to the Parkinson's disease or obstructive detrusor instability.

In the elderly, factors outside the lower urinary tract may be important in causing or worsening incontinence. Attention needs to be directed at the patient's mobility, mood and cognitive state, as well as to their lower urinary tract.

### ASSESSMENT

The bladder is an unreliable witness (*Farrar et al*,1978, *McGuire et al*,1980 *Drutz* and *Mandell* 1979). The different pathophysiological mechanisms leading to incontinence can produce similar lower urinary tract symptoms. Diagnoses based on clinical assessment alone are often wrong. Clinical accuracy is greatest when diagnosing Genuine stress incontinence and worst when diagnosing voiding disorders in females. Accurate diagnosis requires the use of urodynamic studies. However this is not to say that these are a necessary prerequisite to the proper management of all incontinent elderly patients. Apart from measurements of urinary flow rate and residual urine, urodynamic investigations have only a small part to play in the management of incontinent elderly males. They are indicated in patients with outflow obstruction in whom endoscopy fails to reveal any obstructing lesion. Here their value is to demonstrate unobstructed detrusor instability. They are also indicated in male patients with stress incontinence and in some patients with neurological lesions, eg., multiple sclerosis, traumatic spinal cord damage.

The assessment of female patients is more difficult. Using the algorithm prepared by Hilton and Stanton (1981) it is possible to correctly manage over 90% of patients by performing urodynamic studies on 40%. It's main drawback is a failure to identify voiding discorders (*Eastwood* and *Warrell* 1984). Urodynamic studies are indicated on female patients with stress incontinence and other lower urinary tract symptoms, such as frequency, nocturia, urgency or urge incontinence. If practical they should be performed on all patients with stress incontinence prior to surgery. This avoids patients whose stress incontinence is due to detrusor instability having unnecessary surgery. Whether patients with detrusor instability in addition to Genuine stress incontinence should be operated upon is a matter of controversy. Urodynamic studies are also indicated where patients have had failed surgery for Genuine stress incontinence, patients with voiding disorders and patients with neuropathic bladder disorders. Patients with urge incontinence but no stress incontinence are usually assumed to have detrusor instability providing the causes of sensory urgency are excluded. This requires cystoscopy rather than urodynamic studies. Some specialists recommend urodynamic studies for patients with presumed detrusor instability, who fail to respond satisfactorily to treatment.

When dealing with incontinent elderly patients, therapeutic options are often limited by advanced age or the presence of physical or mental morbidity. Urodynamic studies should not be performed unless the result is likely to affect management.

### MANAGEMENT

Many drugs have been introduced for the management of detrusor instability and hyperreflexia. Most are anticholinergics, calcium antagonists or smooth muscle relaxants. Some have more than one potentially beneficial mode of action on the lower urinary tract. Few have stood the test of time. They rarely by themselves, lead to the restoration of continence. Of those currently available Oxybutynin is probably the most effective. It has combined anticholinergic and smooth muscle relaxant properties. It results in a delay in the onset and a decrease in the amplitude of unstable contractions. There is a reduction in the frequency of miturition and in the number of incontinent episodes (*Moore et al* 1990, *Ouslander et al* 1988). Most patients experience side effects which are usually anticholinergic. Around a quarter stop taking the drug because they find the side effects, particularly the dry mouth, intolerable. Little information is as yet available on its efficacy in the elderly. Imipramine may also be worth trying. As a beta stimulant it leads to detrusor relaxation and as an alpha stimulant it may increase bladder outflow resistance. It also has anticholinergic effects. If these agents fail there is little evidence as yet to justify trying the alternative drugs available.

Oestrogens and alpha adrenerergic agents are used in the non surgical management of Genuine stress incontinence. Oestrogens are believed to lead to an increased density and sensitivity of alpha adrenerergic receptors and improvement in the mucosal seal mechanism. Studies to date have failed to show consistent benefit from their use. Alpha adrenerergic agents are effective in mild to moderate stress incontinence. Although well tolerated, they may have significant side effects. The cholinergic agent Bethanechol has been the drug of choice for the promotion of bladder emptying. Although effective invitro, doubt has been cast upon its clinical efficacy (*Finkbeiner* 1985).

Surgery remains the most effective therapeutic intervention for female patients with Genuine stress incontinence and male patients with detrusor instability secondary to outflow tract obstruction. Patients with idiopathic detrusor instability and detrusor hyperreflexia who fail to respond to medical treatment remain a major therapeutic challenge, for which no satisfactory surgical option exists. The surgery of choice for these patients at present is augmentation cystoplasty (*Bramble* 1990). The bladder is almost completely bivalved in the coronal plane. A length of ilium with it's own blood supply is then sutured to the bladder. The nett result is that involuntary bladder contractions are rendered ineffective. The efficacy of voluntary detrusor contractions may also be compromised. Most patients have to strain to void and around a third require intermittent self catherisation. This is a major operation with significant complications. Most experience to date has been with young and middle aged patients. It is unlikely that it will ever have a major place in the management of incontinent elderly patients. None of the different operations developed with the common aim of denervating the bladder have proved useful. The most promising of these, subtrigonal phenol injection, has recently fallen out of favour because of the short duration of any benefit gained (*Rosenbaum et al* 1990).

The most effective therapy for patients with idiopathic detrusor instability is a bladder retraining regime (*Frewin* 1978; *Holmes et al* 1983). This is a psychomedical treatment based on the assumption that the idiopathic unstable bladder is a functional disorder. It's aim is to teach the patient to gain control over her bladder. She is instructed to pass urine at a specified time. She ignores sensations from her bladder and waits until the specified time before voiding. She cannot go earlier. She either resists the urge to void or is incontinent. The interval between voidings is increased until the patient can maintain continence for four hours. Reported cure rates vary from 60 - 80%. Cure is usually associated with the resolution of urodynamic abnormalities. It has no place in the management of patients with organic brain disease.

Patients with detrusor hyperreflexia are best managed using a regular timed voiding regime. No attempt is made to alter bladder function. The aim is to minimise incontinence by having the patient void before the onset of an unstable contraction. This type of voiding regime is the mainstay of the management of incontinence among the elderly in institutional care. More sophisticated voiding regimes have been introduced, eg.Habit Retraining, Prompted voiding and behaviour therapy. The additional benefit gained by using these time consuming voiding regimes, does not justify the additional effort involved.

Physiotherapy is an effective and safe way of managing many female patients with Genuine stress incontinence. Around half the patients are cured or sufficiently improved to decide that they do not require surgery. Some authors have found that the elderly and those with severe stress incontinence respond poorly to physiotherapy. Others have failed to identify any difference in response rates.

If all else fails incontinence may be managed by the use of catheters. This may involve the use of external collecting devices, indwelling catheters or intermittent self catheterisation. The use of indwelling catheters should be the final therapeutic option. All too often it is the first and only treatment used. Long term catheterisation in the elderly is often complicated by significant urinary tract infections, encrustation and catheter bypassing or extrusion (*Sabanathan et al* 1985, *Getliffe et al* 1991).

Management of the incontinent elderly patient needs to be multifaceted. Thus management of an elderly lady with detrusor hyperreflexia secondary to a cerebrovascular event may involve the use of a regular timed voiding regime, Oxybutynin, physiotherapy to improve her mobility, use of appropriate protective garments and environmental manipulation.

### REFERENCES

THOMAS T.M., PLYMAT K.R., BLANNIN J., MEADE T.W. (1980). Prevalence of Urinary Incontinence - *Br. Med. J.:* **281**: 1243 - 1245.

MACAULEY A.J., STERN R.S., HOLMES D.M., STANTON S.L. (1987). Micturition and the Mind: Psychological Factors in the Eteology and Treatment of Urinary Symptoms in Women. *Br.Med.J.*: **294**: 540 - 543.

CUCCHI A. (1988). Detrusor Instability and Bladder Outflow Obstruction. Evidence for a Correlation between the Severity of Obstruction and the presence of instability. *Br. J. Urol.*: **61**: 420 - 422.

FARRAR D.J., WHITESIDE D.G., OSBORNE J.L., TURNER-WARWICK A. (1975). Urodynamic Analysis of Micturition Symptoms in the Female. *Surg.Gynaecol. Obstet.*: **141**: 875 - 881.

McGUIRE E.J., LYTTON B., KOHORAN E.I., PEPE V. (1980). The Value of Urodynamic Testing in Stress Urinary Incontinence. *J. Urol.*: **124**: 256 - 258.

DRUTZ H.B., MANDENLL F. (1979). Urodynamic Analysis of Urinary Incontinence symptoms in Women. *Gynaecology*: **134**: 789 -792.

HILTON P., STANTON S.L. (1981). Algorithmic Method for Assessing Urinary Incontinence in Elderly Women. *Br. Med. J.*: **282**: 940 - 942.

EASTWOOD H.D.H., WARRELL R. (1984). Urinary Incontinence in the Elderly Female: Prediction in Diagnosis and Outcome of management. *Age Ageing*: **13**: 230 - 234.

MOORE K.H., HAY D.M., IMRIE A.E., WATSON A., GOLDSTEIN M. (1990). Oxybutynin Hydrochloride in the Treatment of Women with Idiopathic Detrusor Instability. *Br.J. Urol.*: **66**: 479 - 485.

OUSLANDER J.G., BLAUSTIN J., CONNOR J., ORZECK S., YONG C.L. (1988). Pharmacokinetics and Clinical Effects of Oxybutynin in Geriatric Patients. *J. Urol.*: **140**: 47 - 50.

FINKBEINER A.E. (1985). Is Bethanecol Chloride Clinically Effective in Promoting Bladder Emptying? A Literature Review; *J. Urol.*: **134**: 443 - 448.

BRAMBLE. (1990). The Claim Cystophasty. *Br.J.Urol.*: **66**: 337 - 341.

ROSENBAUM T.P., SHAW J.R., WORTH P.H.R. (1990). Transtrigonal Phenol failed the Test of Time. *Br.J. Urol.*: **66**: 164 -169.

FREWIN W.K. (1978). An Objective Assessment of the Unstable Bladder of Psychosomatic Origin. *Br. J. Urol.*: **50**: 246 - 249.

HOLMES D.M., STONE A.R., BARRY B.R., RICHARDS L.J., STEVENSON T.P. (1983). Bladder Training three years on. *Br.J. Urol.:* **55**: 660 - 664.

GETLIFFE K.A., MULHALL A.B. (1991). The Encrustation of Indwelling Catheter. *Br.J. Urol.*: **67**: 337 - 341.

SABANATHAN K., CASTLEDEN C.M., MITCHELL C.J. (1985). The Problem of Bacteruria with Indwelling Urethral Catheterisation. *Age Ageing*: **14**: 85 - 90.

